# Bridging technology and pedagogy: evaluation of an interprofessional course on technology-enhanced learning for health professions educators

**DOI:** 10.3389/fmed.2026.1832222

**Published:** 2026-06-09

**Authors:** Jason Wen Yau Lee, Nigel Choon Kiat Tan, Fernando Bello

**Affiliations:** 1 Technology Enhanced Learning and Innovation Department, Duke-NUS Medical School, National University of Singapore, Singapore, Singapore; 2 Department of Neurology, National Neuroscience Institute, Singapore, Singapore; 3 Group Chief Education Officer, Singapore Health Services, Singapore, Singapore; 4 Department of Surgery and Cancer, Imperial College, London, United Kingdom

**Keywords:** blended learning, faculty development, health profession education, interprofessional education, Kirkpatrick and Kirkpatrick learning model, technology enhanced learning (TEL)

## Abstract

**Background:**

The proliferation of educational technologies presents both opportunities and challenges for health professions educators. A prior institutional needs assessment found that educators across all professions struggle to adopt these technologies, as they must navigate not only technical requirements but also the pedagogical principles that govern their effective use. This descriptive study evaluated a 4-credit interprofessional Executive Certificate (EC) designed to equip educators with the technological tools and the underpinning pedagogical principles to support their use. The course participants include doctors, nurses, allied health professionals, and education support administrative staff actively involved in teaching and learning within the institution.

**Methods:**

Graduates across 5 years (2021–2025) of the Technology Enhanced Learning for Health Professions Education (TEL4HPE) were invited to complete an online survey that assesses the outcomes at Kirkpatrick Level 2 (self-efficacy), Level 3 (behavior), and Level 4 (results). The survey was designed to measure self-efficacy in TEL competencies, behavioral changes in teaching practice and organizational impact using Likert-scale and slider-scale items analyzed with descriptive statistics, Kruskal-Wallis test and one-way ANOVA.

**Results:**

Seventy-one participants completed the survey (44.1% response rate) from across six professional groups. They reported high confidence in applying educational principles (*M* = 4.13) and using technology for teaching (*M* = 4.07). After completing the program, most respondents reported redesigning their courses (60.6%) or integrating new technology into their teaching practice (57.7%). About 60% of the respondents shared that their institution had adopted the TEL initiatives they introduced. No significant differences were found across cohorts or professional groups, suggesting an equitable impact.

**Conclusion:**

The TEL4HPE program was associated with high self-reported confidence, reported changes in teaching practice, and perceived organizational influence. The interprofessional cohort model provides a promising approach to faculty development for health professions educators, with implications for institutional design and support of TEL initiatives.

## 1 Introduction

### 1.1 Background

With the ever-increasing number of technologies available, educators have a multitude of tools to enhance their teaching. Yet having so many tools can create a paradox of choice (1), where too many options lead to decision paralysis. Educators often struggle to determine which tools and methods would best meet their needs to help learners achieve intended outcomes. A prior institutional needs assessment found that healthcare educators across medicine, nursing, allied health, and administrative roles reported gaps in readiness to teach online and identified specific technology-related training needs, highlighting the demand for structured faculty development in this area (2). The gap between technological availability and pedagogical understanding creates a need for a structured faculty development program that bridges theory and practice (3), particularly in healthcare education, where the rapid adoption of technology-enhanced learning (TEL) has outpaced systematic educator training.

A persistent challenge in TEL is the tendency for educators to transfer traditional face-to-face teaching approaches into the online environment (4, 5). Without formal training, educators often replicate the instructional methods of their own teachers, assuming these practices remain effective regardless of medium (6). However, effective TEL requires intentional design that leverages technology’s affordances to create an authentic learning experience, not simply a digitized version of classroom instructions. Addressing these challenges requires more than just access to tools. Educators will need structured opportunities to develop both the technical and pedagogical competencies, ideally within an interprofessional context that reflects the collaborative nature of healthcare practices.

### 1.2 Rationale and study purpose

While various faculty development programs exist for technology integration, few specifically address the needs of healthcare professions educators, and fewer still bring educators from different professions together to learn collaboratively. Yet contemporary healthcare education increasingly relies on teaching teams that span professional boundaries. An interprofessional approach to faculty development recognizes this reality, enabling educators from the doctor, nursing, dentistry, allied health, and administrative roles to develop shared competencies in TEL.

Evaluating the effectiveness of faculty development programs requires a systematic framework. The Kirkpatrick model (7) describes four levels of training outcomes: reaction (participant satisfaction), learning (changes in knowledge, skills, or attitudes), behavior (application to practice), and results (organizational or system-level impact). While systematic reviews of faculty development consistently report positive outcomes at Levels 1 and 2, evidence at Levels 3 and 4 remains limited, with organizational impact particularly under-examined (8).

Although systematic reviews have investigated technological trends in medical education (9) and faculty development programs aimed at enhancing teaching effectiveness (3), notable gaps persist. Firstly, technology-focused reviews primarily assess the influence of specific tools on student learning outcomes, rather than the development of competencies among healthcare educators. Secondly, evaluations of faculty development efforts (10) tend to focus on Kirkpatrick Levels 1 and 2, with little exploration of outcomes at the organizational level (3, 10). Thirdly, despite advocacy for longitudinal approaches to faculty development, few studies provide multi-year follow-up data demonstrating sustained behavioral change and organizational impact. Finally, to the best of our knowledge, no studies have evaluated a comprehensive, interprofessional technology-enhanced learning (TEL) program specifically designed to develop healthcare educators across various professional groups.

The TEL4HPE Executive Certificate was designed to address this need. The program brings together healthcare educators from medicine, nursing, allied health, dentistry, and administration into shared interprofessional cohorts. Drawing on social constructivism, adult learning theory, and interprofessional education principles, it combines foundational modules in educational theory and TEL design with elective modules in specialized areas such as simulation, immersive learning, and learning analytics. This study evaluates the program logic (11, 12) across five cohorts (2021–2025) and examines outcomes at Kirkpatrick Levels 2, 3, and 4. The study was guided by four research questions:

1. What is the long-term impact of the program on participants’ self-efficacy in TEL?
2. How have participants applied TEL strategies in their teaching practice?
3. What organizational-level outcomes have resulted from participant involvement?
4. How do outcomes compare across time, professional backgrounds, and prior experience?

## 2 Pedagogical frameworks

### 2.1 Learning theories

Social constructivism posits that learning is an active process in which learners construct knowledge through interaction with others and their environment (13). The program applies this principle by emphasizing collaborative activities, peer discussion, and shared problem-solving rather than didactic instruction. Adult learning theory further informed the program design, recognizing that healthcare educators bring prior experience and immediate application needs to their learning (14). Therefore, the modules were designed to incorporate participants’ own teaching contexts as the basis for activities, enabling direct transfer of learning to practice.

### 2.2 Interprofessional education

The program extends IPE principles to healthcare educators by deliberately bringing together doctors, nurses, allied health professionals, dental educators, and administrators into shared cohorts. This design enables participants to exchange perspectives on teaching challenges across different healthcare contexts and learn from each other’s disciplinary expertise. The interprofessional cohort model recognizes that healthcare educators, regardless of professional background, face common pedagogical and technological challenges in their teaching practice.

### 2.3 Instructional approach

The Core modules use a team-based learning (TBL) approach, combining individual preparation with collaborative application exercise (15). This approach promotes accountability, active engagement, and peer learning. Across all modules, practical application is emphasized: participants work with authentic examples from healthcare education and apply concepts directly to their own teaching contexts. This design ensures that learning is immediately relevant and transferable to learners’ workplace settings.

Table 1 summarizes how each theoretical framework is translated into specific course design decisions and instructional activities, and the expected outcomes at each Kirkpatrick level. This mapping illustrates the coherence between the program’s pedagogical foundations and its evaluation framework.

**TABLE 1:** Summary of theoretical framework, design principle, instructional activities and expected outcomes.

Theory	Design principle	Course activity	Expected outcome (Kirkpatrick level)
Social constructivism	Learning through collaborative interaction and shared problem-solving	Team-based learning readiness assurance tests and application exercises in Core modules	Level 2: Improved self-efficacy in applying educational principles through peer-mediated learning
Social constructivism	Community of practice through ongoing peer relationships	Small group discussion within the TBL group and a wider Telegram community	Level 3: Sustained application of TEL strategies supported by peer networks
Adult learning theory	Learners bring prior experience and need immediate relevance	Module activities anchored in participants’ own teaching contexts and authentic healthcare education examples	Level 2: Confidence in applying TEL competencies directly to own practice
Adult learning theory	Self-directed learning with protected reflection time	Mid-module 1-week break designed to allow processing and reflection before the next session	Level 3: Deliberate transfer of learning to workplace teaching practice
Interprofessional education	Cross-professional exchange of teaching perspectives	Interprofessional cohort composition across medicine, nursing, allied health, dentistry, and administration	Level 3: Broader application of TEL across professional contexts
Interprofessional education	Diverse role modeling across professional boundaries	Alumni co-facilitators recruited from multiple professions in the preceding cohort for Core modules	Level 4: Institutional adoption of TEL initiatives introduced by participants from multiple professional groups
Team-based learning	Individual accountability combined with collaborative application	Individual Readiness Assurance Tests (iRAT) followed by Team Readiness Assurance Tests (tRAT) and application exercises	Level 2: Demonstrated learning of TEL concepts through structured individual and group assessment

## 3 Learning environment

### 3.1 Context and participants

The program is offered by Duke-NUS Medical School, Singapore, and taught by the faculty from the Technology Enhanced Learning and Innovation department within the Office of Education. Participants were recruited from across SingHealth’s network of acute hospitals, national speciality centers, community hospitals, and polyclinics (16). The course targeted healthcare educators, including doctors, nurses, allied health professionals, dentists, and healthcare administrators.

From 2021 to 2025,161 participants representing various professions participated in the course (see Table 2). Most notably, doctors and nurses contributed 40 participants, followed by 39 Allied Health professionals. The participation of 23 healthcare administrators began only in 2023, with the establishment of the College of Healthcare Administration and Leadership. Dentistry and Duke-NUS had 8 and 9 participants, respectively, with 2 external participants not affiliated with healthcare education. This composition of interprofessional participants across the cohorts provided a rich and diverse learning experience.

**TABLE 2:** Breakdown of participants from the different professions.

Cohort	Doctor	Nursing	Allied Health	Healthcare Admin	Dentistry	Duke-NUS	External	Total
Cohort 2021	4	6	5	0	1	1	0	17
Cohort 2022	12	11	11	0	1	4	0	39
Cohort 2023	13	13	11	5	2	1	1	46
Cohort 2024	7	6	6	6	2	1	0	28
Cohort 2025	4	4	6	12	2	2	1	31
Total	40	40	39	23	8	9	2	161

### 3.2 Course structure

Each module is worth one course credit or “unit” in the institution. It consists of one weekly 3.5-h lesson running for 3 weeks. We designed all the modules to run for 2 weeks each during a month, with a 1-week break in between (e.g., weeks 1, 2, and 4). This mid-module break was intentionally designed to provide learners with time to reflect on and process their learning experience. For each learning module, learners engage in at least ten contact hours with faculty through a combination of in-person and synchronous online activities. While initially designed for in-person delivery, the two Core modules were delivered online in 2021 due to COVID restrictions, without changes to the learning outcomes.

Core modules employ team-based learning, combining pre-class preparation, individual and team readiness assurance tests, and collaborative application exercises. The core modules, GMS5301 and GMS5302, establish foundational competencies in educational theory and TEL design. Participants then select two elective modules from five options: Fundamentals of Simulation-based Education, Immersive Learning, Serious Games, Learning Analytics, and Implementing Online Assessments. Across all modules, participants apply concepts directly to their own teaching contexts through practical activities and projects. Detailed module descriptions, learning outcomes, and teaching approaches are provided in Supplementary Material 1.

### 3.3 Alumni as co-facilitators

We deliberately recruited previous course graduates as an integral part of the course to co-facilitate the Core Modules alongside the instructor and to foster community among participants. As co-facilitators from various professions, including doctors, nurses, allied health professionals, dentists, and administrative staff, the alumni provide first-hand experience in navigating the challenges of integrating technology into their professional practice. At least two co-facilitators were recruited from the immediate previous cohort for this approach.

The purpose of having diverse co-facilitators was to broaden learners’ understanding of how technology can be applied meaningfully across various professional contexts. They bring specialized knowledge and practical experience within the healthcare setting that the instructor does not possess. For example, a nursing alumna shared the development process and challenges she faced when developing a serious game; her experience provided insights, and her perseverance served as positive role modeling for the other participants. An administrative professional shared how he used learning analytics extracted through the LMS to improve learning. This interprofessional exchange encourages participants to think beyond their discipline to foster a more holistic approach to integrating technology.

Alumni co-facilitators benefited from mentorship on small-group facilitation skills, reinforcing their learning, while the instructor gained insights into TEL applications across healthcare contexts. This partnership modeled the community of practice that the course aimed to develop.

### 3.4 Evaluation design

This study used a cross-sectional design to evaluate the program’s long-term impact. Graduates from all the cohorts were invited to participate in an online survey in January 2026 to assess outcomes at Kirkpatrick Levels 2, 3, and 4. An initial email invitation was sent to all program graduates, followed by reminder emails at 2-week intervals, for a total of 3 contact attempts. Participation in the study was voluntary, and no individual identifiers were collected.

### 3.5 Survey instrument

The survey instrument was developed specifically for this study with items aligned to the program learning outcomes and structured around Kirkpatrick’s evaluation framework. The instrument was reviewed for content validity by two co-authors with complementary expertise: Assoc Prof Nigel Tan, a healthcare educator with over 20 years of experience in health professions education, and Prof Fernando Bello, a professor of technology-enhanced learning and innovation with over 20 years of experience in program and course evaluation across qualitative and quantitative methodologies.

The survey consists of five sections. The first gathers demographic data, including cohort year, professional background, completion of an elective module, and previous TEL experience. The second evaluates confidence in applying the acquired knowledge and skills through a 5-point Likert scale (1 = not confident at all, 5 = very confident). Two items measure Core module competencies, while elective-specific questions are shown only to participants who completed that elective. Due to display-logic inconsistencies in the online survey, response rates to elective-specific confidence items varied. For analysis, elective-specific confidence ratings were restricted to respondents enrolled in the corresponding elective.

The third section explored behavioral changes and current practices, including modifications to teaching, examples of TEL application, usage frequency, and barriers and enablers to implementation. The fourth section evaluated perceived impact using five items rated on a 0–100 slider scale (0 = no impact at all, 100 = very strong impact), covering areas such as technology use in teaching, TEL initiatives introduced, colleague adoption, reputation as an educator, and perceived return on investment. Participants also indicated whether their institution had adopted the TEL initiatives they introduced and provided examples. The fifth section collected future needs and made program recommendations.

The two-item Core-module competency confidence scale showed good internal consistency with a Cronbach’s α of 0.82, and the fourth section’s five-item impact scale also demonstrated strong reliability with a Cronbach’s α of 0.86. Item-total correlations for the impact scale ranged from 0.53 to 0.80, indicating all items contributed significantly to measuring the construct. Open-ended questions encouraged participants to share examples of changes implemented, the impact made, enablers, barriers, and suggestions for future course improvements.

### 3.6 Data analysis

The quantitative data were analyzed utilizing SPSS (version 29 Mac). Descriptive statistics were calculated for all survey items, and Likert-scale confidence items were summarized as means with standard deviations.

Subgroup analysis was conducted to examine whether outcomes varied across the cohorts, professional background, and prior TEL experience (RQ4). One-way ANOVA was used to compare means across the years, while the Kruskal-Wallis test was used to compare professions and prior experience due to the unequal group sizes. Pearson correlations were calculated to examine the relationships between confidence and program impact variables, with p < 0.05 set as the threshold for statistical significance.

Open-ended responses were reviewed by the lead author to identify illustrative examples relevant to each research question, covering reported changes in teaching practice, TEL application, enablers, barriers, and perceived impact. Representative quotes were selected to contextualize the quantitative findings. The co-authors reviewed the selected quotes to verify their representativeness. Given the program evaluation purpose of this study, the qualitative data were intended to illustrate rather than extend the quantitative findings, and no formal coding framework or thematic structure was applied. Of the 71 respondents, 46 provided open-ended comments on changes to teaching practice and 44 described enablers to implementation, providing a pool of illustrative responses.

### 3.7 Ethical consideration

This study was reviewed by the NUS Institutional Review Board and classified as “review not required” (NUS-IRB-2026-171) as it constituted a program evaluation using anonymized data. A waiver of consent was granted under this classification. Participation in the survey was voluntary, and no individual identifiers were collected.

## 4 Results

### 4.1 Respondents demographics

A total of 71 participants responded to the survey, representing a 44.1% response rate (see Table 3). Responses were distributed across all five cohorts and six professional groups. The largest professional groups to respond were Allied Health (31%), healthcare administrators (22.5%), and doctors (21.1%). Most respondents (66.2%) reported prior TEL experience before the program, while 19.7% reported no or minimal experience.

**TABLE 3:** Characteristics of survey respondents.

Characteristics	n	%
Cohort year		
2021	7	9.9
2022	16	22.5
2023	16	22.5
2024	15	21.1
2025	17	23.9
Profession		
Doctor	15	21.1
Nurse	13	18.3
Allied health	22	31
Healthcare admin	16	22.5
Dentistry	3	4.2
Duke-NUS	2	2.8
Prior TEL experience		
None/Inimal	14	19.7
Some (occasional use)	47	66.2
Moderate (regular use)	9	12.7
Extensive (frequent implementation)	1	1.4

### 4.2 Self-efficacy after the program (Kirkpatrick Level 2)

**FIGURE 1:**
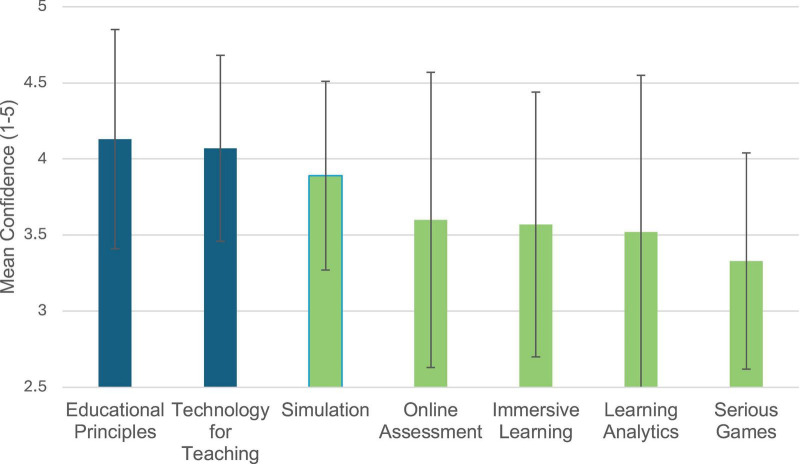
Mean self-efficacy scores by module with standard deviation error bars. Core modules are shown in blue; elective modules in green. n varies by module (Core modules *n* = 67; electives as reported in text).

Participants reported a high level of confidence in applying the Core competencies (see Figure 1). The mean confidence rating for the two core modules was 4.13 (SD = 0.72) for applying educational principles in teaching, and 4.07 (SD = 0.61) for using technology to support teaching needs. Most participants reported feeling confident to very confident in applying educational principles (91.0%) and using technology for teaching purposes (88.1%).

The mean confidence for the electives varied by module and was assessed only among participants enrolled in the respective elective. Simulation-based education had the highest confidence rating (*n* = 36, M = 3.89, SD = 0.62), followed by online assessment (*n* = 10, *M* = 3.60, SD = 0.97), immersive learning (*n* = 21, *M* = 3.57, SD = 0.87), learning analytics (*n* = 33, *M* = 3.52, SD = 1.03), and serious games (*n* = 9, *M* = 3.33, SD = 0.71). The small sample for serious games (*n* = 9 of 36 enrolled) reflects a lower response rate to this item and limits interpretation of this estimate.

### 4.3 Application to teaching practice (Kirkpatrick level 3)

Most participants reported making changes to their teaching practice after completing the program. 60.6% of the participants reported redesigning their course based on pedagogical principles learned, and 57.7% reported integrating new technology into their course (Table 4).

**TABLE 4:** Behavioural change to teaching practice.

Behavioural change	*n*	Total participants	%
Core module changes			
Redesigned course based on pedagogical principles	43	71	60.6
Integrated new technology into course	41	71	57.7
Elective-specific changes			
Applied simulation principles (GMS5311)	22	38	57.9
Used immersive learning technology (GMS5312)	2	23	8.7
Used analytics to handle student data (GMS5314)	12	33	36.4
Used serious games in teaching (GMS5313)	3	36	8.3
Implemented online assessment (GMS5315)	3	12	25.0
TEL tools currently used			
Online polls/quizzes	63	71	88.7
Flipped classroom	46	71	64.8
Elective-specific tools			
Simulation techniques (GMS5311)	25	38	65.8
Immersive learning technologies (GMS5312)	4	23	17.4
Serious games (GMS5313)	8	36	22.2
Learning analytics (GMS5314)	15	33	45.5
Online assessment (GMS5315)	8	12	66.7

For elective-specific behavioral change, simulation-based education had the highest number (57.9%) of participants applying simulation principles to their practice, followed by learning analytics (36.4%) and online assessment (25%). There was a lower application of principles learned in immersive learning (8.7%) and serious games (8.3%).

Regarding the TEL tools used, online polls and quizzes were the most prevalent (88.7%), followed by flipped classroom approaches (64.8%). Among the elective-specific tools, online assessment tools were most frequently adopted (66.7%), followed by simulation techniques (65.8%). In contrast, adoption rates were lower for learning analytics (45.5%), serious games (22.2%), and immersive learning (17.4%).

Open-ended responses illustrated the range of changes made to teaching practice. One participant described applying TEL skills across multiple contexts: “I’ve also since applied the TEL skills into a TBL program—orientation for the new doctors, as well as incorporating some of the elements into an ongoing communication workshop.” Another described a more systematic pedagogical approach: “I applied the ‘principle of constructive alignment’ to strengthen the link between learning outcomes, teaching/workshop activities, and assessments. For the synchronous workshops conducted as TBL sessions, I designed application-focused assessments using case scenarios, which encouraged deeper conceptual understanding.” In some cases, practice changes led to institutional innovation: “Developed the first VR game for nurses in my institution to enhance the code blue readiness and performance of nurses. Game has just been completed and we are working on implementing it.”

When asked about the frequency of TEL strategy use (*n* = 67), 3.0% reported using them rarely (once per year or less), 44.8% occasionally (in some courses), 46.3% often (most courses), and 6.0% in nearly all courses. Percentages do not sum to 100% due to rounding.

### 4.4 Organizational impact (Kirkpatrick level 4)

Participants rated the program’s organizational impact on a 0–100 scale. The mean ratings for using technology for teaching (*M* = 74.8, SD = 14.6) and perceived return on investment (*M* = 74.5, SD = 17.6) were highest. The impact on TEL initiatives introduced (*M* = 66.7, SD = 19.4), reputation as an educator (*M* = 66.0, SD = 18.9), and colleague adoption (*M* = 61.6, SD = 20.7) was comparatively lower, but still reflected an overall positive impact of the program (Table 5).

**TABLE 5:** Impact of the program on the organization.

Impact	*n*	M	SD
Using technology for teaching	54	74.8	14.6
Perceived return on investment	53	74.5	17.6
TEL initiatives introduced	53	66.7	19.4
Reputation as educator	54	66.0	18.9
Colleague adoption	51	61.6	20.7

Of the 56 respondents who answered this item, 58.9% (*n* = 33) reported that their institution had implemented or adopted TEL initiatives that they introduced, while 41.1% (*n* = 23) reported no institutional adoption. Examples of institutional initiatives included: “Online TBL core training and communication workshop,” “Blended learning with instructional videos designed to be engaging,” “Serious gaming in cannulation and infection control practice,” and “Enhanced simulation workshops with new interactive modules and advanced debriefing techniques.”

The program received strong endorsement: 81.5% of respondents indicated they would recommend it to their colleagues, and 18.5% indicated they might recommend it. No respondents said they would not recommend the program.

### 4.5 Subgroup analysis

Subgroup analyses were conducted to examine whether outcomes varied by cohort year, professional background, and prior TEL experience.

No significant differences were found across cohort years in confidence or impact ratings (all p > 0.05), suggesting consistent program quality and sustained impact across cohorts. The follow-up period varied considerably across cohorts, ranging from less than 1 year for Cohort 2025 to approximately 5 years for Cohort 2021, with elective modules only available from 2022 following the resumption of in-person classes after COVID-19 restrictions. Similarly, no significant differences were found across professional groups (all p > 0.05), indicating the program is equally effective for doctors, nurses, allied health professionals, and administrators (Table 6).

**TABLE 6:** Subgroup analysis on confidence and impact by cohort, profession, and prior experience.

Outcome variable	By cohort	By profession	By prior experience
	F(p)	H(p)	H(p)
Confidence			
Applying educational principles	1.15 (0.34)	4.14 (0.529)	1.53 (0.466)
Using technology for teaching	1.34 (0.264)	8.05 (0.153)	6.02 (0.049)*
Impact			
Using technology for teaching	1.02 (0.406)	4.45 (0.487)	3.27 (0.195)
Perceived return on investment	0.56 (0.695)	7.39 (0.193)	5.11 (0.078)
TEL initiatives introduced	0.3 (0.877)	6.97 (0.223)	0.18 (0.913)
Reputation as educator	0.43 (0.789)	4.7 (0.453)	1.77 (0.412)
Colleague adoption	0.49 (0.744)	7.2 (0.206)	0.25 (0.883)

*p < 0.05.

One significant difference emerged for prior experience. Participants with moderate prior TEL experience reported higher confidence in using technology for teaching than those with minimal or some experience (*p* = 0.049). Participants with no or minimal prior experience reported the highest perceived return on investment, though this difference did not reach statistical significance (*p* = 0.078).

### 4.6 Barriers and enablers

The most common barriers to implementing TEL within their own institution were technical limitations (57.7%), resource issues (53.5%), and time constraints (46.5%). A small number of participants reported a lack of institutional support (15.5%) and learner resistance (9.9%).

The enablers identified in open-ended responses included peer and supervisor support, institutional buy-in for TEL platforms, funding for tools, availability of technical support, and having multiple trained colleagues. As one participant noted: “Support from HODs and open-mindedness of the colleagues. About 1/4 of us have attended the TEL course, and we have found it to be very effective.” However, not all participants experienced a successful transfer. One noted limited organizational impact despite personal gains: “My team was already at a certain level so I did not contribute further to what they already possessed. I tried to introduce items like community of enquiry using platforms like Google Classroom but residents rarely responded nor contributed. All busy working and fighting daily battles.” Structural constraints also limited implementation for some: “The overall impact is really muted by internet separation. And Teams meetings really limits interactivity.”

## 5 Discussion and practical implications

These findings provided evidence of perceived positive outcomes at Kirkpatrick Levels 2, 3, and 4, addressing a notable gap in faculty development literature.

### 5.1 Sustained TEL competencies

Participants reported high confidence in applying core TEL competencies, with over 88% in applying educational principles and using technology for teaching (Kirkpatrick Level 2). These findings align with other faculty development studies (8, 10), which reported higher self-efficacy gains after training. More importantly, the absence of a statistically significant difference across the cohort suggests that higher self-reported confidence was maintained over time.

Confidence for elective-specific competencies was somewhat lower, particularly for serious games and immersive learning. This likely reflects the higher barriers associated with these technologies in healthcare education, including limited institutional readiness, high development costs, and resource constraints previously identified among healthcare educators in this context (2). In contrast, the higher confidence reported for simulation-based education likely reflects its more established role in clinical training, where structured simulation programs have been widely adopted.

An unexpected finding emerged from the subgroup analysis of prior experience. Participants with no or minimal prior TEL experience reported the highest perceived return on investment, though this difference did not reach statistical significance (*p* = 0.078). This suggests that the program may offer the greatest added value for novice educators, who have the most to gain from structured TEL training. On the other hand, participants with moderate prior experience reported higher confidence in using technology for teaching, suggesting that prior exposure accelerates competence development within the program. These findings suggest that educators across a range of starting points benefit differently from the program.

### 5.2 Transfer to teaching practice

These findings suggest that a structured faculty development program is associated with self-reported behavioral change (Kirkpatrick level 3) among healthcare professional educators. Many participants indicated they redesigned their courses (60.6%) or incorporated new technology into their teaching (57.7%) after completing the program. These results go beyond standard satisfaction metrics, offering evidence of self-reported changes in teaching practices consistent with the program’s pedagogy-first, practice-based approach.

Simulation-based education showed the highest transfer rate at 57.9%, followed by learning analytics at 36.4%, and online assessment at 25%. The transfer of immersive learning (8.7%) and serious games (8.3%) was much lower, despite moderate adoption of these tools among participants. This lower transfer is consistent with the barriers reported by participants, including technical limitations and resource constraints. These findings suggest that the effective implementation of advanced TEL approaches depends not only on individual competence but also on institutional infrastructure and organizational support, consistent with the faculty development literature.

### 5.3 Organizational impact

One of the most significant findings of this study is the evidence of perceived organizational influence (Kirkpatrick level 4). 58.9% of the participants reported that their institution had adopted the TEL initiatives they introduced. These findings suggest that the program contributed to organizational TEL adoption across 5 years of implementation, though the cross-sectional design and reliance on self-reported data preclude causal attribution.

Examples of institutional adoption included online TBL training programs, blended learning with instructional videos, serious games for clinical skills practice, and enhanced simulation workshops with advanced debriefing techniques.

However, approximately 40% of respondents did not report institutional adoption of their TEL initiatives. This could be linked with the barriers identified in this study, where technical limitations (57.7%), resource constraints (53.5%), and time pressures (46.5%) were the most frequently cited obstacles to implementation. These findings suggest that individual competence gained through training is necessary but not sufficient for institutional adoption. Structural factors, including access to technology, protected time, and organizational buy-in, play a critical role in determining whether individually acquired TEL competencies translate into sustained institutional change. This aligns with broader faculty development literature suggesting that transfer of training is heavily mediated by the work environment.

### 5.4 Equity of impact

The comparable outcomes across professional groups suggest that the interprofessional cohort model delivers equitable impact regardless of professional background, supporting the program’s design intention to develop shared TEL competencies across disciplinary boundaries. This is a particularly meaningful finding given the diversity of participants, spanning medicine, nursing, allied health, dentistry, and administration, who joined from 2023, which may limit the interpretation of their outcomes. Future evaluations should nonetheless seek larger samples from smaller professional groups to enable more robust subgroup comparisons.

### 5.5 Implication for practice

There are several practical implications arising from this study’s findings. Firstly, this study suggests that programs that extend beyond a one-time workshop offer benefits, allowing participants to apply, reinforce, and refine their newly acquired competencies in practice. Second, successful implementation of TEL initiatives appears to depend not only on individual capability but also on institutional infrastructure, technical support, and protected time.

A notable outcome was the emergence of a TEL community of practice (17). Deliberate grouping of learners with varied experiences led to an informal Telegram group that continued beyond the course, serving as a platform for ongoing peer learning (3).

## 6 Conclusion

This study provides evidence that an interprofessional faculty development program in technology-enhanced learning is associated with high self-reported confidence after the program, changes in teaching practice, and perceived organizational adoption across five cohorts of healthcare educators. To the best of our knowledge, this is the first study to evaluate a comprehensive interprofessional TEL faculty development program using Kirkpatrick Levels 2, 3, and 4, addressing a notable gap in the faculty development literature. However, findings should be interpreted in light of the cross-sectional design, reliance on self-reported data, and variable follow-up periods across cohorts, which preclude causal attribution.

Institutions seeking to implement similar programs are encouraged to consider the following recommendations. First, faculty development programs in TEL should extend beyond one-time workshops to allow participants sufficient time to apply, reinforce, and refine their competencies in practice. Second, institutions should allocate protected time and dedicated technical support for educators following training, as individual competence alone is insufficient for sustained TEL adoption without organizational infrastructure. Third, an interprofessional cohort model should be considered as a deliberate design choice, as shared learning across professional boundaries supports the development of a community of practice that extends beyond the program itself.

## 7 Limitations

This study has several limitations that should be considered when interpreting the findings.

Firstly, the cross-sectional survey design relied entirely on self-reported data collected at a single time point, without a pre-program baseline or a comparator group. As a result, it is not possible to attribute changes in teaching practice or organizational adoption directly to the program, and the findings reflect perceived rather than objectively verified outcomes.

Second, we received a response rate of 44%, which raises the possibility of non-response bias, as respondents who had a positive experience may inflate outcome estimates. As noted in section 3.4, demographic data for non-respondents were unavailable, precluding a formal non-response bias analysis.

Third, the survey instrument was developed specifically for this study, demonstrated good internal consistency, but has not been validated in other contexts or by an independent external expert.

Fourth, the follow-up period varied considerably across cohorts, ranging from less than one year for Cohort 2025 to approximately 5 years for Cohort 2021. Findings from more recent cohorts should therefore be interpreted with caution, as participants may have had insufficient time to apply and consolidate their learning.

Fifth, display-logic inconsistencies in the online survey resulted in variable response rates for elective-specific confidence items. The sample for the serious games elective was particularly small (*n* = 9 of 36 enrolled), limiting the reliability of that estimate.

Sixth, the qualitative component was limited to a content review conducted by the lead author, with co-authors verifying the selected quotes. There was no formal coding framework or inter-rater reliability process applied, which may limit the depth and rigor of the illustrative analysis.

Finally, the study was conducted at a single institution in Singapore, which may limit the generalizability to other healthcare contexts.

Despite these limitations, this study provides valuable evidence of the impact of an interprofessional TEL faculty development program, an area where the literature remains scarce.

## Data Availability

The raw data supporting the conclusions of this article will be made available by the authors, without undue reservation.
