# Supplementary material for: Bridging technology and pedagogy: evaluation of an interprofessional course on technology-enhanced learning for health professions educators
Source: Front Med (Lausanne). 2026 Jun 9;13:1832222. doi: 10.3389/fmed.2026.1832222 (PMC13286825; doi:10.3389/fmed.2026.1832222)
Supplement: Supplementary file 1 [file Data_Sheet_1.docx]

**Supplementary Material 1**

**TEL4HPE Executive Certificate: Module Descriptions, Learning Outcomes, and Teaching Approaches**

This supplementary document provides detailed information on the seven modules comprising the Technology Enhanced Learning for Health Professions Education (TEL4HPE) Executive Certificate. For each module, we present the module description, intended learning outcomes, and the teaching approach employed. This information is intended to support readers who wish to understand or adapt the program design for their own institutional context.

**1. Core Modules**

The two core modules aim to build participants' foundations in the principles of teaching and learning, preparing them to integrate technology into their teaching and to foster an engaging learning environment.

**1.1 GMS5301 Principles of Learning and Teaching in the Professions**

***Module Description***

This course provides a framework for developing the skills necessary for course design, teaching, learning, assessment, and facilitating learning within a professional context. Participants build confidence in teaching through reflection and engagement in a community of practice. With their peers, they explore learning theories, course design, and good instructional practices. The course concludes with an opportunity to put theory into practice through a microteaching activity or portfolio, with feedback from peers and experts.

***Intended Learning Outcomes***

By the end of the module, participants will be able to:

- Develop a critical and reflective mindset on learning and teaching in professional contexts
- Design courses for adult learners with explicit outcomes aligned to learning activities and assessments
- Use questioning and facilitation strategies to promote effective learning among students
- Evaluate their teaching to determine the impact of teaching strategies on student outcomes
- Appreciate the value of building a community of practice of educators within their professional environment

***Teaching Approach***

This module uses a team-based learning (TBL) approach, which emphasises preparation outside the classroom and the application of knowledge during in-class sessions. Learners are organised into small groups of no more than six, which remain stable throughout the duration of the module. Before each session, learners complete required readings and the class begins with an Individual Readiness Assurance Test (iRAT), followed by a Group Readiness Assurance Test (gRAT). These assessments contribute to a small component of the final grade to foster accountability and group collaboration. In-class time then focuses on application activities in which teams work together on significant, authentic problems that learners are likely to encounter in their workplace or the next level of study.

**1.2 GMS5302 Principles and Applications of Technology Enhanced Learning**

***Module Description***

This course introduces participants to the principles of teaching and learning with technology. Participants explore, evaluate, and incorporate existing technologies in their own teaching practice to promote and encourage learning. The module provides the theoretical foundation for those who wish to further explore advanced topics in using technology to enhance learning.

***Intended Learning Outcomes***

By the end of the module, participants will be able to:

- Explain how technology can be used within their own practice to encourage and promote learning
- Identify issues and trends in technology for learning
- Analyse existing digital technologies to support current and future learning needs
- Design a technology-enhanced learning activity to achieve the desired learning outcomes

***Teaching Approach***

This module also adopts the team-based learning approach described above, with iRATs, gRATs, and application exercises structured around authentic teaching problems. The application component emphasises hands-on interaction with digital tools, allowing participants to evaluate technologies in the context of their own teaching.

**2. Elective Modules**

The elective modules offer participants the opportunity to select areas of professional interest and need once they have established a foundational knowledge of teaching and learning. All elective classes are held in person at a centrally located campus in Singapore. Class size is limited to 20 participants, and learners rank their elective preferences. We ensure each class includes a mix of learners from different professions, aligning with the spirit of fostering interprofessional collaboration.

**2.1 GMS5311 Fundamentals of Simulation-based Education in Health Professions Education**

***Module Description***

This course exposes participants to the fundamentals of simulation-based education (SBE) in healthcare. It explores the educational theories underpinning SBE, scenario design, the creation of safe learning environments, and the facilitation of immersive simulation, including feedback and debriefing. Participants have the opportunity to design, develop, and implement a simulation-based educational activity specific to their healthcare setting.

***Intended Learning Outcomes***

By the end of the module, participants will be able to:

- Identify the educational theories that inform SBE
- Outline a range of simulation modalities that can be applied to healthcare simulation
- Discuss the advantages and disadvantages of implementing SBE
- Describe the phases of SBE and how the components of each phase impact learning
- Describe appropriate uses of technology for SBE
- Demonstrate how to plan and design a simulation-based learning activity

***Teaching Approach***

Participants engage in role-plays and debriefing sessions grounded in best practices for simulation education. These activities enable learners to practise and reflect on the integration of technology in realistic teaching scenarios, enhancing their ability to apply these skills in real-world clinical settings.

**2.2 GMS5312 Immersive Learning**

***Module Description***

This course provides an introduction to immersive learning, an approach that uses Extended Reality (XR) technologies to create highly interactive virtual or simulated environments in which learners are fully immersed in the learning process. Participants learn about the principles of immersive learning and the key XR enabling technologies. They also learn to recognise the opportunities and challenges presented by immersive learning and to evaluate its use in healthcare education.

***Intended Learning Outcomes***

By the end of the module, participants will be able to:

- Explain the principles of immersive learning
- Identify key enabling XR technologies
- Describe how XR technologies may be used to support immersive learning
- Evaluate immersive learning for healthcare education
- Plan an immersive learning use case

***Teaching Approach***

Participants have hands-on opportunities to use virtual reality (VR) headsets to explore simulated healthcare scenarios within a virtual environment. This experience allows them to critically assess the potential and limitations of VR in teaching within their professional contexts, fostering a deeper understanding of immersive technologies.

**2.3 GMS5313 Serious Games: Application in Healthcare**

***Module Description***

This course introduces participants to the use of games for learning. While games are typically associated with entertainment, “serious games” are designed with a specific learning or behavioural intent. Participants learn the principles of designing games and evaluate the use of serious games for learning and patient care.

***Intended Learning Outcomes***

By the end of the module, participants will be able to:

- Explain the principles of game design
- Describe how games can be used for learning
- Evaluate games for healthcare management
- Plan a serious game for learning

***Teaching Approach***

Participants encounter game-based learning in the context of medical education, gaining firsthand experience with this innovative learning approach. They learn about the principles of game-based design and how these can be applied to create engaging and effective educational experiences. By applying educational theories such as constructivism and experiential learning, participants critically reflect on the pedagogical value of serious games.

**2.4 GMS5314 Learning Analytics and Student Performance**

***Module Description***

Learning analytics uses technology to improve learning by capturing course delivery and student learning data to report on student performance. Through learning analytics, educators can obtain objective data to understand student learning and enable faster iteration to close the loop of teaching, learning, and feedback. In this course, participants learn to design and identify student learning data collection strategies; handle, clean, and curate data; visualise and analyse student performance data; formulate approaches for providing real-time feedback; and develop skills for data-informed decision-making.

***Intended Learning Outcomes***

By the end of the module, participants will be able to:

- Formulate student performance questions
- Design and integrate learning analytics approaches with data collection, analysis, and visualisations into their curriculum to assess student performance
- Adapt technologies and tools in learning analytics into their daily content delivery
- Perform appropriate data handling, cleaning, and curation for detailed analysis

***Teaching Approach***

Using an experiential learning approach, participants manipulate authentic data sets using specialised software, enabling them to better understand how to use data-driven insights to inform their practice and student learning. By actively working with the data, participants develop a deeper comprehension of analytics and engage in reflective practice as they observe the immediate impact of data-driven strategies.

**2.5 GMS5315 Implementing Online Assessments**

***Module Description***

This course introduces healthcare professionals to the fundamental principles in medical education assessment practice. Participants explore the foundations of evidence-based assessment theories and practices most suitable for the healthcare sector. A special focus is the implementation of authentic and valid online assessments, covering both formative and summative online assessments, as well as the appropriate delivery of actionable feedback.

***Intended Learning Outcomes***

By the end of the module, participants will be able to:

- Explain the underlying principles of best assessment practice, including the theories of validity, formative versus summative nature of assessments, test construction, and modes of assessment
- Explain the challenges and opportunities associated with online assessments
- Formulate a scheme of online assessments
- Design and deliver a single robust online assessment tool

***Teaching Approach***

Participants have the opportunity to explore innovative online assessment tools and platforms designed to enhance assessment practices. By implementing an online assessment themselves, participants directly apply what they have learned and gain firsthand experience with the benefits and challenges of digital assessment methods. This practical application is further enriched by peer feedback from different professions, providing varied perspectives that inform and refine their assessment strategies.

**3. Teaching Strategies Across the Programme**

A variety of teaching strategies are employed across the programme, including team-based learning, case-based learning, simulation, experiential learning, and hands-on software use. These strategies are selected to match the learning outcomes of each module and to ensure that participants engage actively with authentic teaching contexts. Common threads across the programme include the use of authentic problems drawn from participants’ own professional practice, structured opportunities for peer learning, and an emphasis on translating learning directly into workplace teaching.
